# Key Components of the Complement Lectin Pathway Are Not Only Required for the Development of Inflammatory Arthritis but Also Regulate the Transcription of Factor D

**DOI:** 10.3389/fimmu.2020.00201

**Published:** 2020-02-21

**Authors:** V. Michael Holers, Anna Borodovsky, Robert I. Scheinman, Nhu Ho, Joseline Ramos Ramirez, József Dobó, Péter Gál, Jared Lindenberger, Annette G. Hansen, Dhruv Desai, Rasmus Pihl, Steffen Thiel, Nirmal K. Banda

**Affiliations:** ^1^Division of Rheumatology, Department of Medicine, University of Colorado Anschutz Medical Campus, Aurora, CO, United States; ^2^Alnylam Pharmaceutical Inc., Boston, MA, United States; ^3^Skaggs School of Pharmacy, University of Colorado Anschutz Medical Campus, Aurora, CO, United States; ^4^Research Centre for Natural Sciences, Institute of Enzymology, Budapest, Hungary; ^5^Department of Biochemistry and Molecular Genetics, University of Colorado Denver Anschutz Medical Campus, Aurora, CO, United States; ^6^Department of Biomedicine, University of Aarhus, Aarhus, Denmark

**Keywords:** liver, gene silencing, complement, arthritis, MBL-associated serine proteases, Factor D

## Abstract

The complement system plays an important role in the pathogenesis of rheumatoid arthritis (RA). Besides driving lectin pathway (LP) activation, the mannan-binding lectin (MBL)-associated serine proteases (MASPs) also play a key role in regulating the alternative pathway (AP). We evaluated the effects of N-acetylgalactosamine (GalNAc)-conjugated MASP-1 and MASP-2 duplexes *in vitro* and in mice with and without arthritis to examine whether knockdown of MASP-1 and MASP-2 expression affects the development of arthritis. GalNAc-siRNAs for MASP-1 and MASP-2 demonstrated robust silencing of MASP-1 or MASP-2 at pM concentrations *in vitro*. To evaluate the impact of silencing in arthritic mice, we used the collagen antibody-induced arthritis (CAIA) mouse model of RA. Mice were injected a 10 mg/kg dose of GalNAc-siRNAs 3x s.q. prior to the induction of CAIA. Liver gene expression was examined using qRT-PCR, and protein levels were confirmed in the circulation by sandwich immunoassays and Western blot. At day 10, CAIA mice separately treated with MASP-1 and MASP-2 duplexes had a specific reduction in expression of liver MASP-1 (70–95%, *p* < 0.05) and MASP-2 (90%, *p* < 0.05) mRNA, respectively. MASP-1-siRNA treatment resulted in a 95% reduction in levels of MASP-1 protein in circulation with no effect on MASP-2 levels and clinical disease activity (CDA). In mice injected with MASP-2 duplex, there was a significant (*p* < 0.05) 90% decrease in *ex vivo* C4b deposition on mannan, with nearly complete elimination of MASP-2 in the circulation. MASP-2 silencing initially significantly decreased CDA by 60% but subsequently changed to a 40% decrease vs. control. Unexpectedly, GalNAc-siRNA-mediated knockdown of MASP-1 and MASP-2 revealed a marked effect of these proteins on the transcription of FD under normal physiological conditions, whereas LPS-induced inflammatory conditions reversed this effect on FD levels. LPS is recognized by Toll-like receptor 4 (TLR4), we found MBL not only binds to TLR4 an interaction with a K_d_ of 907 nM but also upregulated FD expression in differentiated adipocytes. We show that MASP-2 knockdown impairs the development of RA and that the interrelationship between proteins of the LP and the AP may extend to the transcriptional modulation of the FD gene.

## Introduction

Rheumatoid Arthritis (RA) is a chronic inflammatory autoimmune disease of the joints, affecting ~0.24% of the entire world population (1). The mortality rate for patients with RA is higher than the general population (2), and the substantial morbidity of this condition impacts the public health care system substantially (3). Although new therapeutics have improved outcomes and quality of life, and more patients will gain access to new biosimilars, ~40% patients only respond partially or not at all to the best of current therapies (4). Furthermore, disease-modifying anti-rheumatic drugs (DMARDs) ameliorate the disease but also lead to an increased risk of infections due to immune suppression (5). Despite clinical improvement afforded by these therapeutics, the radiological damage in RA can continue over time due to an underlying inflammatory process (6) and treatment with anti-TNF antagonists, despite clinical improvement, has little effect on diagnostic and pathologically relevant markers like anti-citrullinated protein antibodies (ACPAs) (7, 8).

Citrullinated peptides are considered a prime example of an autoantigen in RA, and the presence of ACPAs is significantly correlated with radiological damage (6). Autoantibodies which develop against these epitopes, also designated anti-cyclic citrullinated peptide (CCP) antibodies, can circulate to deposit in damaged joint tissues, which are rich in citrullinated proteins. However, the development of ACPA alone is not sufficient to trigger RA. For example, it has been shown recently that transgenic (Tg) mice expressing IgM with the V region of an anti–CCP mAb cloned from a RA patient, thus from a naturally generated anti-CCP Ab, failed to develop arthritis (9), a finding consistent with the hypothesis that anti-CCP Abs alone are not the cause of disease. Rather, a “second hit” has been postulated to be required for localization of this immune response to the joint (10–12). The nature of this “second hit” may be quite variable; involving infection or injury and could be provided by innate immune components, including those of the complement system.

We and others have extensively probed the role of the complement protein network in RA pathogenesis to identify targets within the pathway that are suitable for therapeutic development (11, 13–18). Inhibition of complement in virtually every RA mouse model tested leads to decreased disease progression (12). The evidence for complement's role in RA is also evident when examining human patients. Examination of synovial fluid from RA patients shows the presence of complement fragments, indicative of complement activation (19). Blood levels of certain complement activation fragments are also increased in these patients, while levels of full length complement proteins may be decreased (20–22). Clinical trials with complement C5-directed therapeutics such as PMX53 and Eculizumab have so far been unsuccessful in the treatment of RA (18). However, these unsuccessful trials likely reflect the complexity of the complement system and requirement to also block upstream at C3 activation, or the relatively late timing of intervention in the disease course, rather than the lack of a role for complement in RA pathogenesis.

The complement system is a liver-produced but serum-effector killing system of the innate immune system. It has now been recognized to play an important and vital role in many autoimmune diseases. It exists and functions in each and every tissue of the body including in the synovium of joints. The system facilitates clearance of circulating immune complexes, damaged tissues, apoptotic cells, apoptotic bodies and dead cells (23, 24), thus possessing strong anti-inflammatory properties for the benefit of the host, e.g., in the absence of a mature adaptive immune response. Overall, complement is composed of many soluble proteins and receptors and is generated in abundance by the liver (25). Other cells or tissues, however, can secrete complement proteins, including epithelial and endothelial cells as well as adipocytes (26, 27). The complement system is activated by three pathways, i.e., classical pathway (CP), lectin Pathway (LP) and alternative pathway (AP) (28). All three pathways of the complement system are activated by different molecules but converge at the cleavage of C3 and C5, generating C3a, C3b, C5a, and C5b, via C3 and C5 convertases, respectively. C3a and C5a function as activators of inflammatory cells via the C3a and C5a receptors (C5aR) while C5b promotes the assembly of the membrane attack complex (MAC, C5b–C9).

The CP consists of C1q, C1r, C1s, as well as C4 and C2, and the LP consists of pattern recognition molecules (PRMs), including mannan binding lectin (MBL), ficolins (FCNs) and Collectins (CLKs), and the effector proteases, MBL-associated serine proteases (MASPs) MASP-1, -2, and -3. Binding of LP PRMs to carbohydrates on the surface of microorganisms leads to MASP-1 autoactivation, MASP-1-mediated activation of MASP-2, and cleavage of C4 and C2 (29). The CP can't activate C3 and subsequently the AP amplification loop without C4 (30, 31). However, the LP can activate C3 and the AP through bypass routes without the direct involvement C4 or C2 (32). This is only possible through the direct activation of C3 by LP proteases (33).

The *MASP-1* gene encodes three variants via alternative splicing; two variants are MASP-1 and MASP-3 with different serine protease domains, and the third variant is MAp44 (a.k.a. MAP-1), which lacks a serine protease domain (34, 35). Most of the MASP-1 is produced by the liver (36) and it has been shown to influence LP activity through direct activation of MASP-2 (37). The observation that MASP-1/3 cleaves profactor D (proFD) to mature factor D (FD) has created a paradigm shift regarding the direct role of LP proteases in also activating the AP of the complement system (38, 39). Mice lacking MASP-1/3 have no LP and have suboptimal AP activity (38, 39) and these mice are resistant to arthritis (40). Of note *MASP-1/3*^−/−^ mice are also lean (36) suggesting a link between adipose tissue and MASPs. Mice lacking MASP-1 have no LP but have intact AP while mice lacking MASP-3 have no AP but have intact LP (41). Initially it was shown that in patients with 3MC syndrome, with combined MASP-1 and MASP-3 deficiency, the AP was functional while the LP was totally non-functional (37). Later on it was confirmed both in mice and humans that it is MASP-3 and not MASP-1 which cleaves proFD (36, 42–45). These findings were partly confirmed in a 3MC patient lacking only MASP-3, as it was shown that the majority of FD was present as proFD. However, this study also highlighted that in humans minor levels of mature FD are still generated *in vivo* by a MASP-3-independent mechanism. MASP-1 is required for AP activation on certain surfaces, and it was reported that MASP-1 is essential for LPS-induced but not for zymosan-induced AP activation (46). Overall, it is now clear that MASP-3 is a major regulator of the AP. The clinical relevance of inhibition of MASP-3 has recently been highlighted, as targeted RNAi of MASP-3 in the liver of mice with MASP-3 duplex attenuated collagen antibody induced arthritis (CAIA), a mouse model dependent on the AP (36). This study also showed that rMASP-3 cleaved proFD into mature FD *in vivo* in *MASP-1/3*^−/−^ mice (36).

MBL-associated serine protease-2 (MASP-2) was first identified using liver cDNA library (47). Later, an alternative splice variant of MASP-2 was identified as MBL-associated plasma protein of 19 kDa (MAP19) [a.k.a. MAP-2 or small MAP (sMAP)] (48). sMAP was cloned and it has the signal peptide similar to MASP-2 but it has a unique C-terminal sequence and lacks the serine protease catalytic domain (49). MASP-2 can cleave C4 and C2 (50), while MASP-1 cannot cleave C4 but can cleave C2 (51). MASP-2 can also activate the AP through a C4/C2 bypass route that leads to MASP-2-dependent activation of C3 by an unknown mechanism (30, 52). Thus, in the complete deficiencies of C4 or C2 this bypass pathway can still activate the AP as seen in guinea pigs lacking C4 (53) and lupus patients lacking C2 (54).

Overall, liver predominately generates MASP-1 and MASP-2 (55), and they can, therefore, be directly targeted by liver-directed RNA interference (RNAi) mechanisms, such as asialoglycoprotein receptors (ASGPR)-dependent uptake of N-acetylegalactosamine (GalNAc)-conjugated small interfering RNAs (siRNAs) (36). The ASGPR, is conserved between species expressed on all hepatocytes, and binds the tri-antennary GalNAc ligand with nanomolar affinity, resulting in internalization of the receptor and delivery of the GalNAc-conjugated siRNA into the hepatocyte (56, 57). Liver-directed targeting of MASP-3 by GalNAc-conjugated MASP-3-siRNA significantly attenuated arthritis by 50% in mice (36). Therefore, for this current study we used GalNAc-MASP-1-siRNA and GalNAc-MASP-2-siRNA duplexes targeting hepatocytes to examine their effect on arthritis.

Our first objective in this study was to generate and test if GalNAc-conjugated MASP-1 and MASP-2 duplexes silence MASP-1 and MASP-2. Our second objective was to target liver MASP-1 or MASP-2 by RNAi and to test their efficacy *in vivo* in mice with and without CAIA. Our third objective was to explore the mechanism(s) by which liver-directed silencing of MASP-1 and MASP-2 effected the LP and AP systemically. In those studies we found evidence that components of the LP also affect the AP by regulating the transcription of FD. Our hypothesis is that liver derived MASP-2 but not MASP-1 provides the “second hit” and it might be essential for the AP-dependent joint damage, and its targeted intra-hepatic inhibition can lead to the attenuation of arthritis by MBL-MASP-2-dependent regulation of FD, which is predominately generated by adipocytes.

## Materials and Methods

### *In vitro* Selection of GalNAc-siRNA-MASP-1 and GalNAc-siRNA-MASP-2 Duplexes

siRNA sequences were designed based on the available bioinformatics information to target the MASP-1 splice variant of the mouse *MASP1* gene (22 siRNAs) or the *MASP2* gene (46 siRNAs). GalNAc-siRNA conjugates were synthesized using solid phase synthesis methods as previously described (36, 58).

### psiCHECK2-Dual-Glo® Luciferase *in vitro* Assay for MASP-1 and MASP-2 Gene Silencing

The sequence of interest (MASP-1 or MASP-2) was cloned into the multiple cloning region (XhoI-NotI sites) located downstream of the Renilla STOP codon in the 3'UTR. The psiCHECK2-Dual-Glo system enables detection of siRNA-mediated silencing of target sequences fused to a *Renilla* luciferase reporter gene. RNAi-mediated cleavage and degradation of the fusion mRNA can be measured by a loss in *Renilla* signal following siRNA treatment. The psiCHECK2 vector also contains a second reporter gene, Firefly luciferase, which is driven by a different promoter and allows for normalization of *Renilla* expression. An African Green monkey Cos-7 cell line was used *in vitro* to examine the effect of MASP-1 and MASP-2 siRNA duplexes on the expression of MASP-1 and MASP-2 in the psiCHECK2 system. All transfections were repeated for a total of three times. Inhibitory Concentration (IC50) of each MASP-1 or MASP-2 duplexes were calculated from the expression curve and is included on the plots.

### Selection of the Active GalNAc-MASP-1 and GalNAc-MASP-2 siRNA Duplexes *in vivo*

The effect of MASP-1 and MASP-2 duplexes on MASP1 and MASP2 expression was initially characterized in 8 weeks old C57BL/6J mice (Charles River Laboratories). Single doses of 10 mg/kg of MASP-1 and of MASP-2 duplexes were administered via a subcutaneous injection. Livers were harvested on Day 7 post s.q. injection. mRNA was isolated using RNeasy 96 Universal tissue kit (Qiagen). High-Capacity cDNA Reverse Transcription Kit (Applied Biosystems) was used to convert the mRNA samples into cDNA. Levels of MASP-1 and MASP-2 expression were analyzed using qRT-PCR reagents compatible with Roche Lightcycler 480 based on previously published studies (36). The MASP-1 and MASP-2 mRNAs were measured using a customized TaqMan assay (ThermoFisher) and normalized to the endogenous GAPDH expression (ThermoFisher).

### Effect of GalNAc-MASP-1 and GalNAc-MASP-2 Duplexes on Induction of Collagen Antibody-Induced Arthritis in Mice

WT mice were injected s.q. with 10 mg/kg on days −10, −5, and on day 0 either with GalNAc-Luciferase-siRNA (*n* = 10) or GalNac-MASP-1 (*n* = 10) or with GalNAc-MASP-2-siRNA (*n* = 10). Then CAIA was induced in these WT mice by using a mixture of 5 mAb to bovine collagen type II (CII) (Arthrogen-CIA, Chondrex) according to published studies (59–61). All WT mice received i.p., injections of 8 mg of Arthrogen on day 0 and 50 μg of LPS from *E. coli* strain 0111B4 on day 3. All mice were sacrificed at day 10 post anti-collagen antibodies injection. The total duration of the CAIA experiment was day 20 post the first siRNA injection. The clinical disease activity (CDA) in all groups of WT mice was determined every day by two trained laboratory personnel acting independently and blinded as to treatment according to our previously published methods (36, 40, 61, 62).

### qRT PCR to Measure Liver Expressed MASPs in Mice With and Without Disease

Mouse liver from first cohort of WT mice (*n* = 30) injected (s.q.) with GalNac-Luciferase (*n* = 10), GalNAc-MASP-1 (*n* = 10) and GalNAc-MASP-2 (*n* = 10) siRNAs with disease, sacrificed at day 20, were homogenized using a bullet blender (36). The expression of MASP-1 and MASP-2 were measured from the liver by qRT-PCR in a blinded fashion. Similarly, in a second cohort, liver from WT mice (*n* = 34) treated with GalNAc-conjugated Luciferase siRNA (*n* = 8) or with MASP-1 siRNA (*n* = 8) or with MASP-2 siRNA (*n* = 9) or with MASP-1 plus MASP-2 siRNAs (*n* = 9) without disease were processed for RNA extraction. In this study, out of 34 mice, LPS (50 μg/mouse/i.p.) was injected, at day 10, in mice treated with GalNAc-conjugated Luciferase siRNA (*n* = 4) or with MASP-1 siRNA (*n* = 4) or with MASP-2 siRNA (*n* = 5) or with MASP-1 plus MASP-2 siRNAs (*n* = 5) and these mice were sacrificed at day 14. The remaining 16 mice, i.e., 4 mice per treatment group, were not injected with LPS and sacrificed at day 25. Total RNA from the liver was isolated from the liver homogenates using RNAeasy Mini kit (Qiagen Inc., Germantown, MD). 18S ribosomal RNA (18S rRNA) was used as an internal control in each experiment and all data were expressed in pg/ng mRNA/18S RNA. All mRNA samples were analyzed in duplicate. All sample were analyzed by amplifying at 40 cycles according to the methods published previously in Nature Protocols (14, 63). All qRT-PCR data were analyzed by using cDNA based on the standard curve made by using liver RNA from a normal WT mice.

### Time-Resolved Immunofluorometric Assay to Measure MASP-1 Protein

The absolute levels of MASP-1 protein were measured by using Time-resolved immunofluorometric assay (TRIFMA), a sandwich-type immunoassay using europium-labeled detecting agents as described (64). In this method, the concentrations of MASP-1 protein in the sera from WT mice injected with GalNAc-Lucifease-siRNA or GalNAc-MASP-1-siRNA or GalNAc-MASP-2-siRNA were determined by using microtiter wells coated with monoclonal anti-mouse MASP-1 antibody, followed by incubation with samples and detection using biotinylated anti-MASP-1 antibody and subsequently by adding Eu3+-conjugated streptavidin and reading of signal by time-resolved flourometri (Perkin Elmer, Hvidovre, Denmark) (64).

### C4b Deposition on Mannan Particles Using Mouse Serum

To assess the functionality of the LP, a 96-well ELISA Costar plates was pre-coated with 9 ug/ml of mannan diluted in 0.05 M Sodium carbonate buffer, pH 9.5 for overnight at 4°C. The ELISA plates were flipped gently with no wash followed by blocking with 1% BSA 1xPBS (with Ca^2+^ Mg^2+^) for 1 h at room temperature. After washing 3x with 1xPBS 0.05% Tween 20, 100 μl of 10% serum diluted in MBL binding buffer [20 mM Tris, 1 M NaCl, 0.05 % (v/v) Triton X-100, 10 mM CaCl_2_, 15 mM NaN_3_, 1 mg/ml HSA, pH 7. 4] was added to each well in duplicate, and the ELISA plates were left at 4°C overnight. Serum was subsequently removed by washing, and recombinant human C4 (rHuC4) protein (Complement Technology Inc., Tyler, Texas) diluted in sodium barbital buffer was added to the wells. One percent sera from WT and *MASP-2*^−/−^ mice were used as a positive and negative controls, respectively. No target control contained no rHuC4. ELISA plates were incubated for 1.5 h at 37°C followed by 3x washings with 1xPBS 0.05% T20 (with Ca^2+^ Mg2^+^). After 1 h anti-C4C biotinylated antibody (1 μg/ml) diluted in 1xPBS 0.05% T20 (with Ca^2+^ Mg^2+^) and incubated at room temperature for 2 h. One hundred microliter of Streptavidin (dilution 1:1,000) was added to each well after washing three times (with Ca^2+^ Mg^2+^). The ELISA plates were developed using 100 μl of 1:1 diluted Super Signal™ West Pico Plus Chemiluminescent Substrate (Thermo Scientific, Rockford, IL). The reaction was stopped using 50 μl with 2N H_2_SO_4_ and absorbance read at 450 nm, correcting for background at 550 nm.

### Western Blot Analysis for MASP-2

To detect the presence or absence of MASP-2 protein, before, during and after MASP-2 silencing, by Western blot analysis, a total of 40 μL of D-Mannose-Agarose beads (resin) (Sigma) were equilibrated in wash buffer containing TBS, 5 mM CaCl_2_ + 1 mM Pefabloc SC (Sigma, 76307). Ten μL of serum was mixed with 20 μL of wash buffer, added to the resin, and incubated for 1.5 h. The beads were washed three times, re-suspended in 50 μL of wash buffer, and transferred to Pierce Microspin column (Thermo Scientific). The spin columns were centrifuged for 2 min at 4,000 × g, until the resin become dry. Elution of MBL-MASP-2 complexes was performed by adding 30 μL of electrophoresis sample buffer to each tube [7.5 μL of NuPAGE LDS sample buffer (Life technologies, NP0007) + 3 μL NuPAGE reducing agent (Life technologies, NP0009) + 19.5 μL of ddH_2_O] and centrifuging the resin until it is dry. Then 30 μL of the eluate was heated at 80°C for 5 min and loaded on the 10% SDS gel using 1xMOPS (Invitrogen) and 1xNupage (Invitrogen) running buffer. Proteins were transferred to a PVDF membrane using 1xNupage tran (Invitrogen) transfer buffer, and the membrane was blocked in PBS + 5% milk for 2 h. The blot was incubated with a primary antibody, biotinylated monoclonal Rat anti-human MASP-2/MAp19 (1 μg/ml) (6G12, cross reacting with mouse MASP-2) in PBS + 5% milk and then incubated at 4°C overnight (65). HRP-conjugated Streptavidin (diluted 1:1,000) was used as a secondary antibody. Finally, the blot was developed by incubating with 12 mL of SuperSignal West Pico PLUS Chemiluminescent Substrate (ThermoFisher) for 10 min. The blot was scanned using Genegnome XRQ Chemiluminescence Imaging System and Gene Tool analysis software from Syngene (Frederick, MD), and the density of each band was quantified using the Quantity One® software (Bio-Rad, Hercules, CA).

### Measurement of Expression of AP Complement Components in Liver After Silencing of MASPs and LPS Treatment

We measured, by qRT PCR, the expression of complement C3, factor B (FB), factor D (FD), Properdin, C5, MASP-1, MASP-2, and MASP-3 from the liver of mice treated with GalNAc-Luciferase siRNA, GalNAc-MASP-1 siRNA, GalNAc-MASP-2 siRNA, and GalNAc-MASP-1 plus MASP-2 siRNAs. The expression of the above-mentioned complement components were measured according to our published methods (14, 36).

### Western Blots for FD Protein

To determine the increase or decrease in FD protein in the circulation of WT mice, with and without LPS, after silencing with GalNAc-MASP-1/MASP-2 duplexes simultaneously, Western blots for FD were performed according to our published methods (36, 40).

### Determination of the Binding Affinity Between TLR4 and MBL Using Microscale Thermophoresis

Recombinant Human TLR4 (R&D Systems) was re-suspended in a buffer containing 20 mM Tris-HCl pH 7.5, 150 mM NaCl, 5 mM CaCl_2_, and 1 mM BME. TLR4 was fluorescently labeled using the NanoTemper Monolith His-Tag Labeling Kit RED-tris-NTA 2nd Generation kit (MO-L018) per the manufacturer's protocol. TLR4 was titrated with recombinant MBL (clinical grade) with concentrations varying from 20.8 μM to 635 pM, while keeping the labeled TLR4 at 5 nM. Samples were loaded into standard capillaries, and Microscale thermophoresis (MST) experiments were carried out using a Monolith NT.115 pico (NanoTemper Technologies GmbH, Munich, Germany). The thermophoresis was monitored at 20% LED power and high MST power. Data were analyzed and fit to the Kd model using MO. Affinity software (version 2.3) (NanoTempet Technologies, CA) in duplicate.

### Determine Expression of TLR4 and FD in 3T3 Cell Line and Adipocytes

To detect TLR4 on the cell surface of cells by flow cytometry, PE-conjugated mouse anti-TLR4 antibody (CD284) (Biolegend) was used along with matched isotype control, rat IgG2a k PE-conjugated (BioLegend). Primary adipocytes derived from adipose tissue of C57BL6 mice were also examined by flow cytometry. The Mouse 3T3 L1 embryonic cell line was used to examine the expression of TLR4 receptors and FD by qRT PCR as mentioned above. 3T3L1 cells were cultured based on our previously published study (66). 3T3L1 cell line was used as analogous to adipocytes as these cells differentiate into adipocyte-like morphology under certain conditions (66). Differentiated 3T3L1 cells were also stimulated with various doses of LPS (*E. coli*) (1.25 or 2.50 or 5 μg/ml) or with recombinant human MBL (10 or 20 μg/ml) for 48 h to determine their effect on the expression of FD and TLR4.

### Statistics

Some of the TRIFMA data were analyzed by using the Excel program. Analysis of variance (ANOVA) was used to comparing more than two independent groups. Two-tailed Student's *t*-test was used to find out the differences between two means. DATA were expressed as standard error of the mean (SEM). The level of significance was defined as *p* < 0.05.

## Results

### IC50 of GalNAc-MASP-1 and MASP-2-siRNA Duplexes

**Figure 1:**
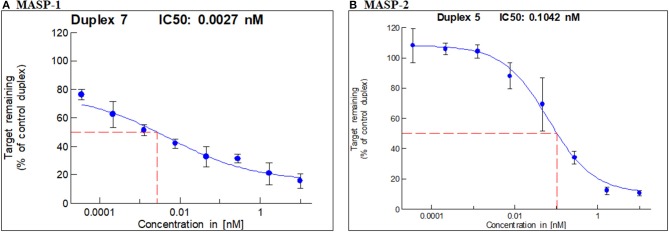
IC50 for MASP-1 and MASP-2 silencing with GalNAc-MASP1 or GalNAc-MASP-2 siRNAs: A Dual-Glo luciferase assay was performed using COS7 cells expressing individually Firefly Luciferase-MASP1 or MASP2 fusion constructs. COS7 cells were transfected with mouse MASP-1 or mouse MASP-2 specific siRNAs at the concentrations indicated. Luminescence levels were measured at 48 hrs post transfection with siRNAs. The ratio of normalized luminescence was plotted in MS Excel to calculate the IC50 for MASP-1 and MASP-2 duplexes— **(A)** MASP-1 duplex IC50 = 0.0027 nM, **(B)** MASP-2 duplex IC50 = 0.1042 nM.

*In vitro* screening led to the selection of one duplex each of GalNAc-MASP-1 (IC50 = 0.0027 nM) and MASP-2 (IC50 = 0.1042 nM) (Figures 1A,B) siRNA. GalNAc-MASP-1-siRNA or GalNAc-MASP-2-siRNA were both 23 bp duplexes. The duplex of GalNAc-MASP-2-siRNA have no off-target effect on MASP-1, and vice versa, as shown below (**Figure 4**).

### GalNAc-MASP-1 and MASP-2 Duplexes Silenced MASP-1 and MASP-2 Expression in the Liver of Mice Without Disease

**Figure 2:**
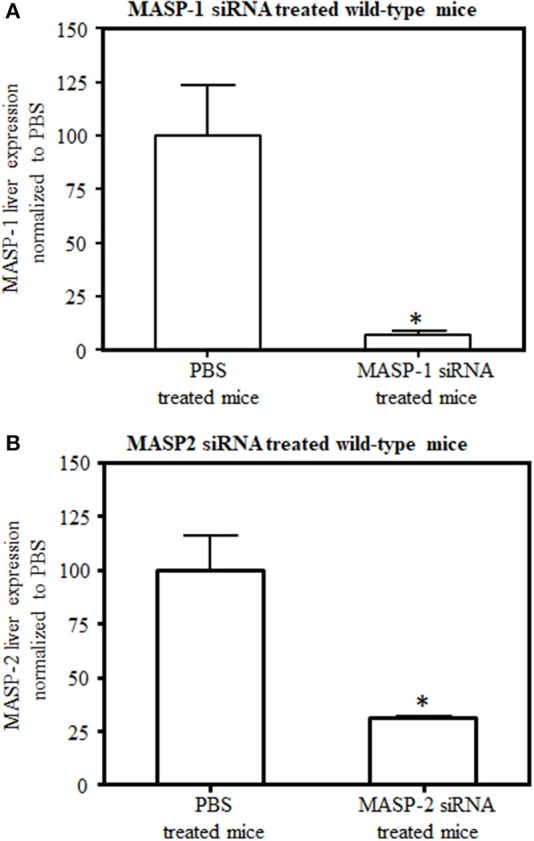
Comparing levels of **(A)** MASP-1 and **(B)** MASP-2 expression by qRT-PCR in C57Bl/6J mice liver. Liver mRNA was extracted at day 7 after mice were injected with either GalNAc–MASP-1–siRNA or GalNAc-MASP2-siRNA or PBS. Gene expression was examined using qRT-PCR. GAPDH expression was used as an internal control to calculate the expression of MASP-1 or MASP-2. Data shown as mean and SEM of three replicates. The *p*-values were calculated using *t*-test. *p < 0.05 vs. PBS.

To determine the *in vivo* efficacy of GalNAc-MASP-1-siRNA and GalNAc-MASP-2-siRNA duplexes on the expression of MASP-1 and MASP-2, healthy C57Bl/6 mice were used (Figure 2). In this experiment, mice were injected with a single dose (10 mg/kg) of either PBS or GalNAc-MASP-1-siRNA or GalNac-MASP-2-siRNA duplexes on day 0 and sacrificed at day 7. A robust silencing in the siRNA-treated groups was observed, i.e., 93% of MASP-1 (Figure 2A) and 69% of MASP-2 (Figure 2B) expression was silenced after a single subcutaneous administration. Initially 58% decrease in MASP-3 expression was also noticed with silencing of MASP-1 but none with MASP-2 silencing.

### GalNAc-MASP-2-siRNA Partially Attenuated Clinical Disease in Mice With CAIA

**Figure 3:**
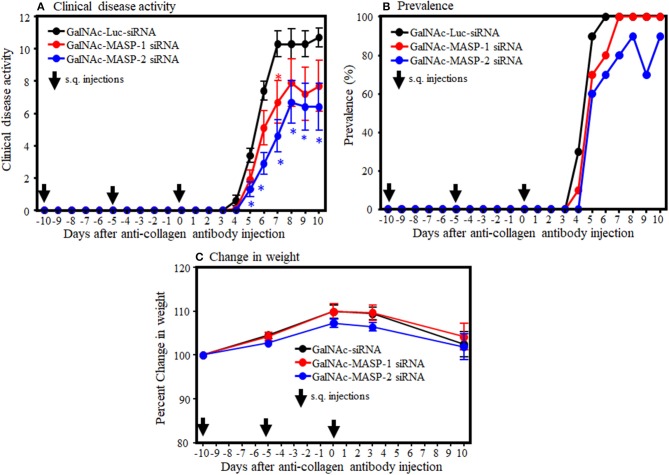
Partial decrease in the clinical disease activity in WT mice treated with GalNAc-MASP-2-siRNA but not with GalNAc-MASP-1 duplex. GalNAc-Luciferase-siRNA was used as negative control compared to GalNAc-MASP-1-siRNA or GalNAc-MASP-2-siRNA. Arthritis, in C57BL/6 mice, was induced by injecting a mixture of five anti-collagen monoclonal antibodies as described in the Methods. WT mice were injected three times s.q with Luciferase siRNAs as a negative control or with GalNAc-MASP-1-siRNA or GalNAc-MASP-2-siRNA at day −10, 0 and at day 3. Disease in mice was checked from day 4 to 10. **(A)** CDA in mice treated with GalNAc-Luciferase-siRNA or GalNAc-MASP-1-siRNA or GalNAc-MASP-2-siRNA. **(B)** Prevalence (%) of disease, in mice treated with GalNAc-Luciferase-siRNA or GalNAc-MASP-1-siRNA or GalNAc-MASP-2-siRNA. **(C)** Change in weight (%) in mice treated with GalNAc-Luciferase-siRNA or GalNAc-MASP-1-siRNA or GalNAc-MASP-2-siRNA. Data shown represent the mean ± SEM based on WT mice injected s.q. with GalNAc-Luciferase-siRNA, *n* = 30 and with GalNAc-MASP-1-siRNA (*n* = 10), GalNAc-MASP-2-siRNA (*n* = 10). *p < 0.05 in comparison to mice to GalNAc-Luciferase-siRNA injected mice (*n* = 10).

To examine the systemic effect of GalNAc-MASP-1-siRNA and GalNAc-MASP-2-siRNA on arthritis, mice were injected (s.q.) three times with GalNAc-Luciferase, GalNAc-MASP-1-siRNA, or GalNAc-MASP-2 siRNA before the induction of disease. All mice injected with anti-CII mAb and LPS developed disease after day 3. At day 10, the CDA in mice injected with GalNAc-Luciferase, GalNAc-MASP-1, or GalNAc-MASP-2 duplexes were 10.7 ± 0.597, 7.7 ± 1.57, and 6.4 ± 1.44, respectively (Figure 3A). In mice treated with GalNAc-MASP-1-siRNA, there was a significant 35% decrease (*p* < 0.030) in the CDA at day 7, whereas there was no difference at day 10 (Figure 3A). However, in mice treated with GalNAc-MASP-2 siRNA there was from day 5 to 10 a consistent decrease in the CDA. At day 10, there was a significant (*p* < 0.013) 40% decrease in the CDA of mice treated with GalNAc-MASP-2-siRNA compared with GalNAc-luciferase, and at day 7 there was a significant (*p* < 0.0003) 60% decrease in the CDA in mice treated with GalNAc-MASP-2 siRNA compared with the mice treated with GalNAc-Luciferase-siRNA (Figure 3A).

The prevalence of disease at day 10 in WT mice injected with GalNAc-Luciferase, GalNAc-MASP-1 and GalNAc-MASP-2 duplexes was 100, 100, and 90%, respectively (Figure 3B). There was no significant (*p* < 0.68) and (*p* < 0.88) effect on the weights of mice treated with GaLNAc-MASP-1 and GalNAc-MASP-2 siRNA throughout the study (Figure 3C). No toxicity was seen, and no mouse died during the course of this experiment. Overall these data suggest that GalNAc-MASP-2, but not GalNAc-MASP-1 partially attenuated CAIA in mice.

### Robust Downregulation of MASP-1 Protein by Liver-Specific RNAi Targeting With GalNAc-MASP-1 Duplex in Mice With CAIA

**Figure 4:**
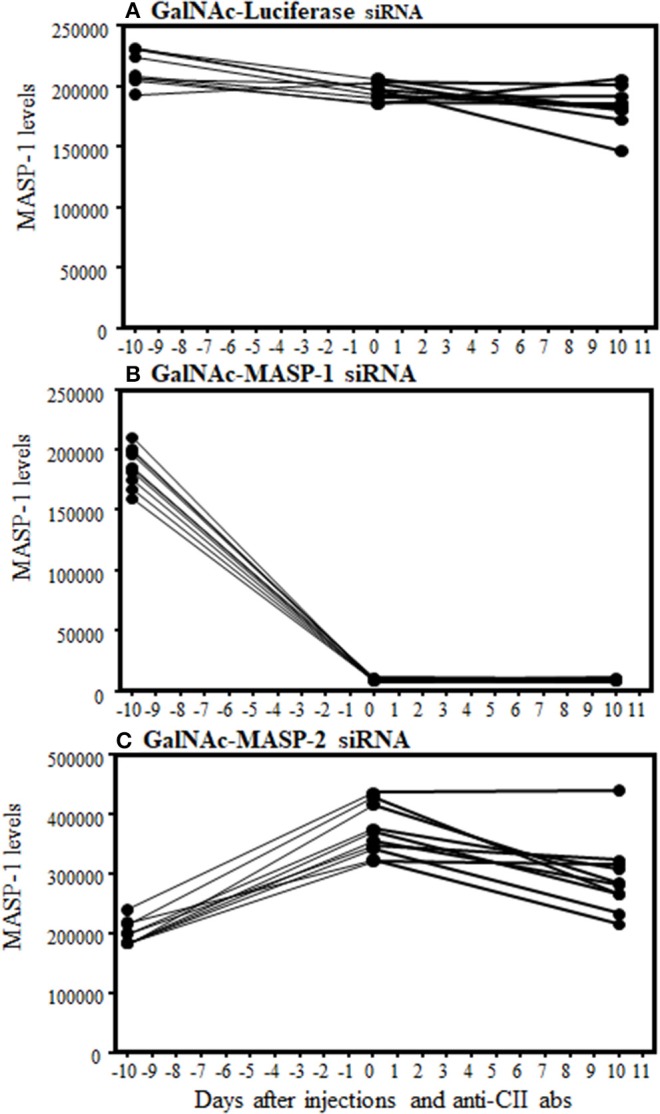
Time-resolved immunoflorometric assay for MASP-1 levels showing a decrease in the absolute levels of MASP-1 protein in the circulation of mice arthritic injected with GalNAc-MASP-1-siRNA. Sera were diluted 1:75 from arthritic mice treated with GalNAc-Luciferase-siRNA, GalNAc-MASP-1-siRNA and GalNAc-MASP-2-siRNA and levels of MASP-1 were examined by ELISA. **(A)** No change in MASP-1 levels at day −10, 0 and at day +10 treated with control GalNAc-Luciferase-siRNA. **(B)** A substantial decrease in the levels of MASP-1 at day 0 and at day +10 vs. −10 in the circulation of mice treated with GalNAc-MASP-1-siRNA. **(C)** No decrease in the levels of MASP-1 at day 0 and at day +10 vs. −10 in the circulation of mice treated with GalNAc-MASP-1-siRNA. Data from all mice (*n* = 30) have been shown. *p* < 0.05 at day 0 and at day +10 vs. at −10 mice injected with GalNAc-Luciferase-siRNA (*n* = 10) or GalNAc-MASP-1-siRNA (*n* = 10) or GalNAc-MASP-2-siRNA (*n* = 10).

A sandwich type immunoassay, i.e., a Time-resolved immunofluorometric assay (TRIFMA) to measure the absolute levels of MASP-1 was used in the sera from mice from the CAIA model (Figure 4). MASP-1 protein levels were measured at baseline (−day 10 relative to disease induction with anti-CII mAbs), after three injections of siRNA (day 0) and after the induction of disease (at day 10) (Figures 4A–C). The levels of MASP-1 were significantly (*p* < 0.0001) decreased by 95% in mice after three injections with GalNAc-MASP-1 siRNA, relative to the mice injected with the GalNAc-Luciferase siRNA, before the development of disease (day 0) and at the end of the study (day 10). (Figures 4A–C). In mice injected with control GalNAc-Luciferase-siRNA or GalNAc-MASP-2-siRNA there was no effect on MASP-1 levels (Figures 4A,C). These data show that MASP-1 duplex specifically and significantly silenced MASP-1 levels systemically for a long time not only before the development of arthritis but even after the development of arthritis. Despite the robust suppression of MASP-1 level, MASP-1 does not play a significant role in the pathogenesis of CAIA, because mice treated with GalNAc-MASP-1-siRNA were not protected as compared to mice treated with control GalNac-Luciferase siRNA (Figure 3A).

### Complete Elimination of MASP-2 Protein by Western Blot Analysis After Liver Targeted RNAi MASP-2 Silencing in Mice With CAIA

**Figure 5:**
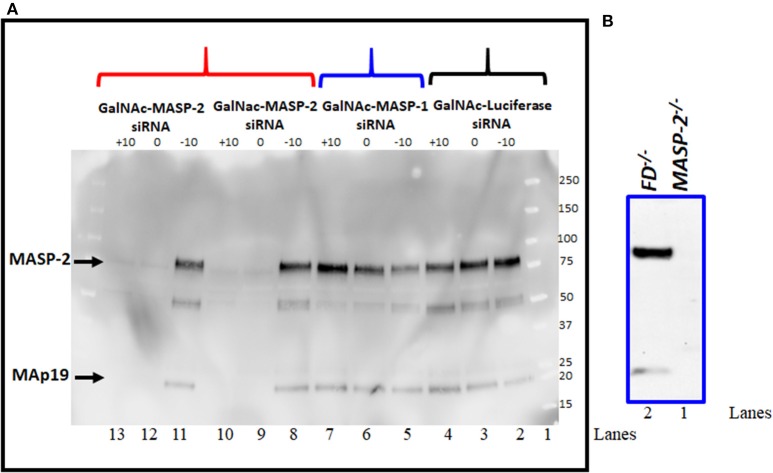
**(A)** Western blot analysis showing nearly complete elimination of MASP-2 in the circulation of WT arthritic mice treated with GalNAc-MASP-2-siRNA. MASP-2 protein from arthritis mice before (day –day 10) and after treatment and before disease induction (day 0) and after disease induction (day +10) was pulled out by using D-Mannose-Agarose beads as mentioned in the Materials and Methods. The primary detection antibody biotinylated Rat anti-human MASP-2/MAp19 (6G12) and secondary antibody HRP-conjugated Streptavidin were used, respectively. Complete absence of MASP-2 (~75 kDa) in the circulation of WT mice treated with GalNAc-MASP-2-siRNA at day 0 and at day +10 (lanes 12, 13). Presence of MASP-2 prior to the treatment with GalNAc-MASP-2 and prior to the disease induction (lane 11). Repeat data from another mouse treated identically with GalNAc-MASP-2-siRNA (lanes 8, 9, and 10). Presence of MASP-2 in the circulation of mice treated with GalNAc-MASP-1-siRNA at day −10, at 0 and at +10 (lanes 5, 6, and 7). Presence of MASP-2 in the circulation of mice treated identically with control GalNAc-Luciferase-siRNA at day −10, at 0 and at +10 (lanes 2, 3, and 4). Lane 1 (protein marker). A band of MAp19 (a.k.a. sMAP) (19 kDa) was also seen in the sera from mice (lanes 2–8 and 11). Sera from *MASP-2*^−/−^ and *FD*^−/−^ mice with no disease were used a negative and positive controls, respectively (**B** lanes 1 and 2). Representative data from GalNAc-Luciferase-siRNA (*n* = 1) or GalNAc-MASP-1-siRNA (*n* = 1) or GalNAc-MASP-2-siRNA (*n* =2) treated mice have been shown. Western blot were repeated 2x with identical results.

Western blot analysis for MASP-2 protein was done to examine the effect on MASP-2 on MASP-1 silencing by GalNAc-MASP-2-siRNA on circulating protein levels (Figure 5). Sera from a subset of mice evaluated in the CAIA model were examined by western blot (Figure 5A). We found that there was no effect on MASP-2 levels in the sera from each mouse injected with GalNAc-Luciferase-siRNA (Figure 5A, lanes 2, 3, 4) or GalNAc-MASP-1-siRNA (Figure 5A, lanes 5, 6, 7). A distinct band of ~75 kDa of MASP-2 was present along with ~19kDa MAp19 (Figure 5A, lanes 2–8 and 11, lane 1, marker). These MASP-2 or MAp19 bands were present only in the sera from mice before treatment GalNAc-MASP-2-siRNA (day −10; Figure 5A, lane 8) but completely absent or barely visible after treatment with GalNAc-MASP-2-siRNA (day 0; Figure 5A, lane 9) and after disease development (day 10; Figure 5A, lane 10). Identical results were seen using serum from another mouse (Figure 5A, lanes 11, 12, 13). Sera from *FD*^−/−^ and *MASP-2*^−/−^ mice were used as positive and negative controls, respectively, to show the specificity of Western blot analysis for MASP-2 protein (Figure 5B, lanes 1, 2). MASP-2 protein is absent in serum from *MASP-2*^−/−^ mouse (Figure 5B, lane 1) as expected but present in the serum from *FD*^−/−^ mouse (Figure 5B, lane 2). These data show that liver targeting of MASP-2 by GalNAc-MASP-2-siRNA was highly effective, and this effect lasted systemically for at least 10 days after the last siRNA dose. Our data once again confirmed that most of the MASP-2 in the circulation is generated by the liver and it can be inhibited below threshold detection levels even during acute inflammation (Figure 5).

### Liver-Targeted RNAi With GalNac-MASP-2 Duplex Robustly Silences C4b Deposition by the LP

**Figure 6:**
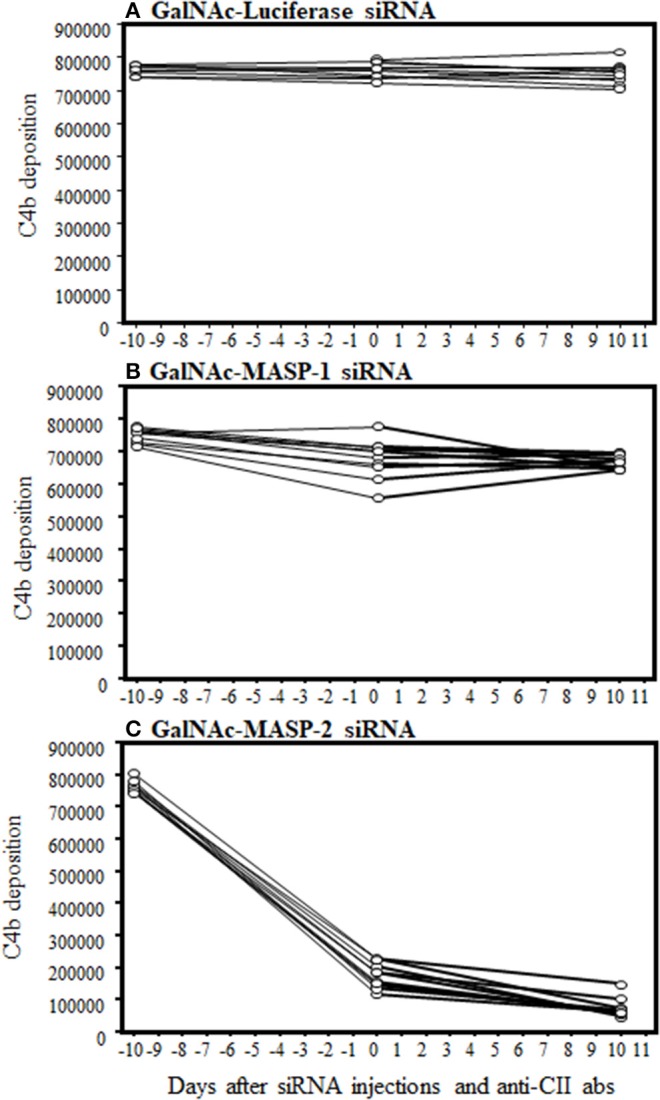
ELISA showing a large decrease in C4b deposition on mannan in the sera from arthritic mice treated three times with GalNAc-MASP-2-siRNA but not with GalNAc-MASP-1-siRNA or GalNAc-Luciferase-siRNA. Sera from mice treated with MASPs or control siRNAs as mentioned above were diluted 1/500 in a tris/high salt/calcium buffer (inhibit any activation by the classical or the MBL pathway) examined at day-10 (before siRNAs injection & before disease induction), day 0 (after injection but before disease induction) and at day 10 (after disease induction). Human recombinant C4 protein was used to show the cleavage into C4b by MASP-2 (mouse MASP-2 bound as MBL/MASP-2 complexes onto the mannan efficiently cleaves human C4—and C4b becomes bound) followed by detection with anti-C4 antibody as mentioned in the Materials and Methods. No change in C4b deposition in the sera from arthritic mice treated with **(A)** GalNAc-Luciferase-siRNA **(B)** GalNAc-MASP-1-siRNA. **(C)** A substantial decrease in C4b deposition in the sera from mice treated GalNAc-MASP-2-siRNA. Data from all individual mice treated with GalNAc-Luciferase-siRNA (*n* = 10) or GalNAc-MASP-1-siRNA (*n* =10) or GalNAc-MASP-2-siRNA (*n* =10) have been shown. *p* < 0.05 at day 0 and at day +10 vs. at −10 mice injected GalNAc-Luciferase-siRNA, GalNAc-MASP-1-siRNA and GalNAc-MASP-2-siRNA.

To examine the functionality of MASP-2 silencing in liver by GalNAc-MASP-2-siRNA, C4b deposition via MBL pathway of the complement on mannancoated microtiter well surfaces was measured by ELISA. This procedure specifically excluded the activation from the CP due to the buffer composition, i.e., no C1q binding occurs to the surface. There is sufficient MASP-1 to activate MASP-2 in GalNac-MASP-1-siRNA treated mice. Sera from mice treated with a single dose of GalNAc-Luciferase-siRNA, GalNAc-MASP-1-siRNA, or GalNAc-MASP-2-siRNA were added to ELISA plates pre-coated with mannan (Figures 6A–C). There was a complete and significant inhibition of C4b deposition both before and after the induction of disease (*p* < 0.05) in the sera from mice injected with GalNAc-MASP-2-siRNA (Figure 6C). These data were remarkably consistent in all mice (10 out of 10) treated with GalNAc-MASP-2-siRNA (Figure 6C). On the other hand, there was no significant effect on C4b deposition from mice treated identically with GalNAc-Luciferase-siRNA or GalNAc-MASP-1-siRNA neither before nor after induction of disease (Figures 6A,B). Overall, these data show that liver-targeted MASP-2 silencing by GalNAc-MASP-2-siRNA completely abrogated C4b deposition (10 out of 10 mice) in the circulation and indicated that most of the MASP-2 is generated by the liver (Figure 6C). Furthermore, MASP-2 duplexes functionally impaired the lectin pathway of the complement. There was no significant off-target effect on C4b deposition of GalNAc-Luciferase-siRNA or GalNAc-MASP-1-siRNA (Figure 6).

### GalNAc-MASP-1 and MASP-2 Duplexes Silenced MASP-1 and MASP-2 Expression in the Liver Mice With Disease

**Figure 7:**
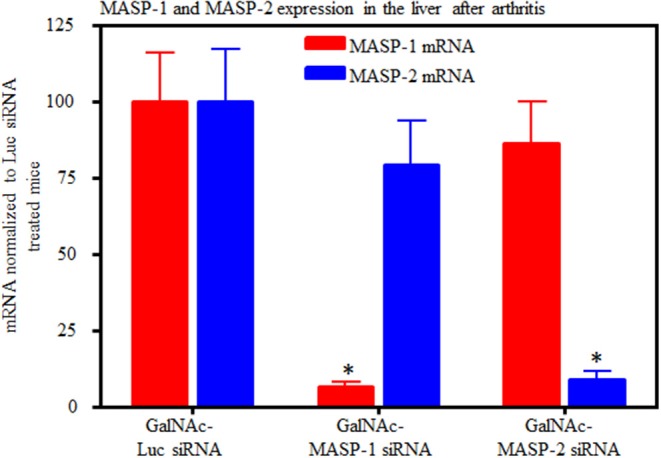
Quantitative RT-PCR analysis of MASP-1 and MASP-2 expression from the liver of WT arthritic mice injected with GalNAc-Luciferase-siRNA or GalNAc-MASP-1-siRNA or GalNAc-MASP-2-siRNA duplexes (*n* = 10 per group). Total RNA was extracted at day 10 from the liver of mice injected s.q. with multiple doses of GalNAc-MASP-1-siRNA or GalNAc-MASP-2-siRNA duplexes. MASP-1, MASP-2, and MASP-3 expression was examined using qRT-PCR. Liver from WT mouse was used as a positive control. Endogenous GAPDH mRNA expression was used as an internal control to normalize the expression of MASP-1 and MASP-2. *p < 0.05 vs. mice injected with GalNAc-Luciferase-siRNA.

**Figure 8:**
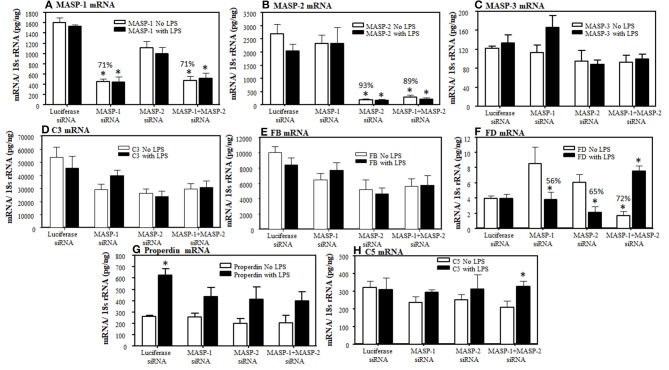
Effect of a long-term silencing of MASP-1 and MASP-2, *in vivo* with no disease, on expression of various AP complement components induced by LPS. The expression of AP complement components was measured by qRT-PCR. WT mice were injected with three doses of GalNAc-Luciferase-siRNA, GalNAc-MASP-1-siRNA, GalNAc-MASP-2-siRNA and GalNAc-MASP-1+MASP-2-siRNAs. At day 10, 3 mice from each group were injected with LPS and sacrificed at day 17 and 2 non-injected control mice were sacrificed at day 25. Liver from all mice examined for the expression of MASP-1, MASP-2, MASP-3, C3, FB, FD, Properdin, and C5. **(A)** MASP-1 **(B)** MASP-2 **(C)** MASP-3. **(D)** C3 **(E)** FB, **(F)** FD, **(G)** Properdin, and **(H)** C5. 18S rRNA was used as internal control and the values have been shown in pg/ng 18S rRNA. *p < 0.05 compared with mice treated with or without LPS. Liver from mice treated with or without LPS were cut into three pieces and the expression of complement components was examined separately and repeated two times from each piece. Total mice used in this study were 34 i.e., GalNAc-Luciferase-siRNA (*n* = 8), GalNAc-MASP-1-siRNA (*n* = 8), GalNAc-MASP-2-siRNA (*n* = 9) and GalNAc-MASP-1+MASP-2-siRNAs (*n* = 9). Data are expressed as Mean ± SEM *p <0.05 considered significant.

Liver expression of MASP-1 and MASP-2 mRNA was examined in mice from the CAIA model. qRT PCR data from the liver of these mice at day 10 confirmed that there was a significant (*p* < 0.05) decrease in the expression of MASP-1 (70–95%) and MASP-2 (90%) with no off target effect on each other (Figure 7). However, in there was a decrease (58%) in expression of MASP-3 in the liver from normal mice with arthritis treated with GalNAc-MASP-1-siRNA (data not shown) but not with GalNAc-MASP-2-siRNA (data not shown). Later on we have not seen any effect on MASP-3 under normal physiological or mild pro-inflammatory conditions (Figure 8C). These data were analyzed in a blinded fashion using two different probes and primers by qRT PCR and results were identical.

### Effect on the Expression of Alternative Pathway Components in Liver After Targeted RNAi Silencing of Individual and Combined MASP-1 and MASP-2 Genes

**Figure 9:**
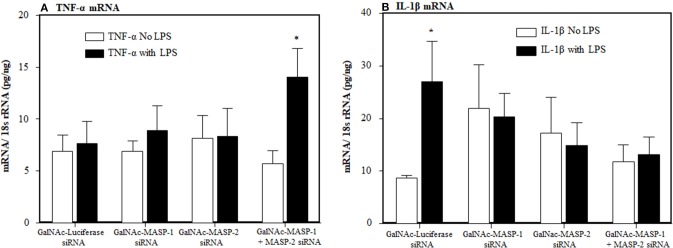
Effect of LPS on inflammatory cytokines after a long term silencing of MASPs. The expression of IL-1β and TNF-α cytokines, in liver, were measured by qRT-PCR. WT mice were injected three times with a single dose of GalNAc-Luciferase-siRNA, GalNAc-MASP-1-siRNA, GalNAc-MASP-2-siRNA and GalNAc-MASP-1+MASP-2-siRNAs followed by an injection of LPS or no LPS injection. Total mice used in this study were 34 i.e., GalNAc-Luciferase-siRNA followed by LPS (*n* = 4) or no LPS (*n* = 4), GalNAc-MASP-1-siRNA followed by LPS (*n* = 4) or no LPS (*n* = 4), GalNAc-MASP-2-siRNA LPS (*n* = 4) or no LPS (*n* = 5) and GalNAc-MASP-1+MASP-2-siRNAs LPS (*n* = 4) or no LPS (*n* = 5). Data are expressed as Mean ± SEM. *p < 0.05 considered significant.

To examine the individual and combined effects of MASP-1 or MASP-2 duplexes on the AP components mice were injected again three times (at day −10, −5, and at day 0) with a single dose (10 mg/kg) of GalNAc-Luciferase-siRNA or GalNAc-MASP-1 siRNA or GalNAc-MASP-2 or GalNAc-MASP-1 + MASP-2 –siRNAs. No disease was induced in these mice but a single dose of LPS was injected at day 10 to generate mile pro-inflammatory conditions. No significant differences in the liver were seen in the expression of C3, FB, and C5 (Figure 8). Surprisingly a significant (*p* < 0.05) decrease of 72% was seen in the liver expression of FD, in mice without injection of LPS, after combined silencing of MASP-1 and MASP-2 (Figure 8F). This reduction in FD expression was reversed in mice injected with LPS (Figure 8F). MASP-2 silencing alone resulted in a reduction of FD mRNA in LPS treated mice (Figure 8F). LPS induced mild inflammatory response as confirmed by slightly enhanced expression of IL-1β and TNF-α cytokines (Figures 9A,B). The expression of Properdin was also significantly (*p* < 0.05) increased in the liver of mice injected with GalNAc-Luciferase-siRNA followed by an injection of LPS but not without LPS (Figure 8G). The decrease in FD expression in the liver show that MASP-1 and MASP-2 together or MASP-2 alone might be regulating the transcription of FD in adipocytes present in the liver in a steady state, but during inflammation this regulating capacity might be lost or switched to something else.

### FD Levels in the Circulation Increase After Simultaneously Silencing of MASP-1/MASP-2 in Response to the LPS

**Figure 10:**
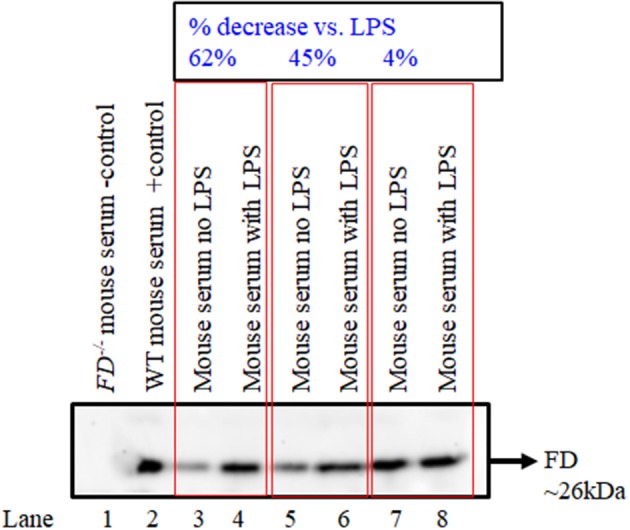
Western blot analysis for FD in serum showing the effect of simultaneously silencing of MASP-1/MASP-2 genes in WT mice followed by treatment with or without LPS. Sera from *FD*^−/−^ and WT mice were used as a negative and positive controls, respectively (lanes 1 and 2). Sera from WT mice with no treatment or with treatment with LPS (lanes 3 vs. 4); (lanes 5 vs. 6) and (lanes 7 vs. 8) after MASP-1/MASP-2 silencing have been shown. The original Western blot was cut to show the actual results for FD. A band of FD ~26 kDa shows the presence of FD. Data from six mice have been shown.

Western blot data show that there was qualitative increase in the levels of FD in the circulation of mice injected with LPS after silencing of both MASP-1/MASP-2 simultaneously, in contrast to the mice without injection of LPS (Figure 10). These data were consistent in six different mice injected with no LPS or with LPS after silencing both MASP-1/MASP-2 genes (Figure 10, lane 3 vs. 4; lane 5 vs. 6 and lane 7 vs. 8). Nonetheless there was variability (4 to 67%) regarding an increase in the levels of FD in the circulation of mice after silencing MASP-1/MASP-2 simultaneously (Figure 10). Again these FD Western blot data in the circulation confirms the mRNA expression data in the liver that combined silencing of MASP-1 and MASP-1 in the liver also effected the FD protein levels in the circulation.

### Binding Affinity of MBL to TLR4 by Microscale Thermophoresis

**Figure 11:**
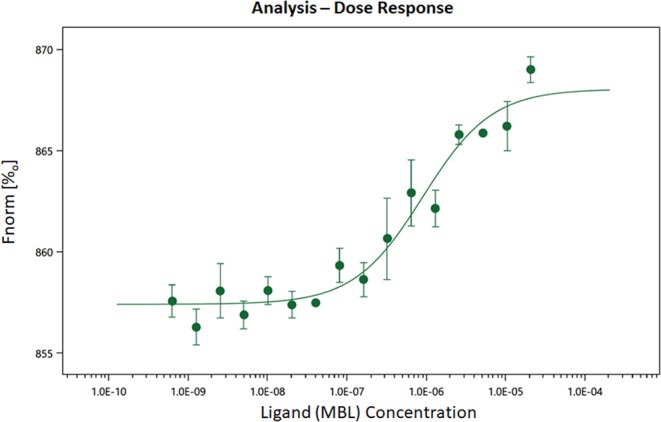
Microscale thermophoresis showing biophysical analysis of binding of human rMBL with human rTLR4/MD2. MST is based on the detection of a temperature-induced change in fluorescence of rTLR4/MD2 (target) as a function of the concentration of a non-fluorescent ligand (rMBL). By titrating MBL into the labeled TLR4 the K_d_ (dissociation constant) was 9.07 × E^−07^ indicating strong binding.

MBL has been previously shown to interact with TLR4 (67, 68), we sought to determine whether the effects of silencing might be through this pathway. In order to confirm the reported binding of MBL to TLR4, MST experiments were carried out. Human TLR4 was fluorescently labeled and titrated against varying concentrations of MBL (Figure 11). All MST traces appeared normal and there was no aggregation (data not shown). The final Kd for the binding was determined to be 907.28 ± 262.77 nM (Figure 11). Thus, we confirm that MBL binds TLR4 directly.

### Expression of TLR4 and FD and in 3T3 Cells and Adipocytes

**Figure 12:**
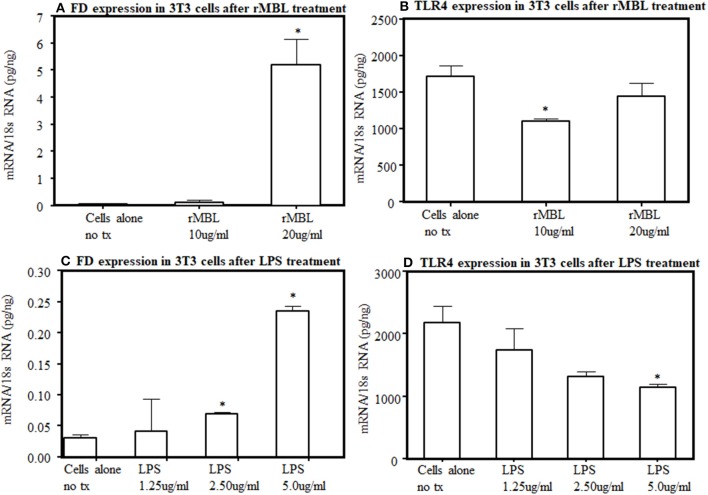
Effect of human rMBL or human rTLR4 on FD expression on differentiated 3T3-L1 cells at 48 h. **(A)** rMBL increased FD expression in a dose-dependent manner. **(B)** rMBL also effected the TLR4 expression. **(C)** LPS also increased FD expression dose-dependent manner. **(D)** LPS also decreased TLR4 expression with increasing doses. Data are shown as Mean ± SEM of three replicative experiments. *p < 0.05 considered significant.

To further understand the interaction between lectin pathway components with LPS activation, we examined the interaction between MBL and TLR4. By flow cytometry, after several repetitions, we found no distinct expression of TLR4 receptors on the surface of 3T3L1 cells as well as from the primary adipocytes derived from WT mice (data not shown). We could not rule out that a small level of TLR4 undetectable with this method is present on their surface. Nonetheless, the endogenous expression of FD and TLR4 at the mRNA level was present in 3T3L1 differentiated adipocytes (Figure 12). There was a clear significant (*p* < 0.006) increase in the expression of FD in differentiated 3T3L1 cells 48 h after stimulating with 10 μg/ml and 20 μg/ml of recombinant MBL but only significant (*p* < 0.05) decrease in TLR4 expression was seen with 10 μg/ml not with 20 μg/ml of recombinant MBL (Figures 12A,B). There was a dose-dependent increase in the expression of FD in differentiated 3T3L1 cells (*p* < 0.05) treated with LPS (Figure 12C). In contrast, a decreasing trend in the expression of TLR4 was seen in response to the LPS (Figure 12D). A correlation (*r* = 0.51) between FD and TLR4 expression in 3T3 cells in response to LPS (5 μg/ml) was noticed but it was not significant (Figures 12C,D). These data indicate although TLR4 is undetectable by flow cytometry on the surface of 3T3 cells but still it can bind to the MBL and its ligand, LPS and modulate FD expression (Figures 12A,D). These data suggest that MBL or LPS can bind to a common receptor TLR4 to regulate the transcription of FD in adipocytes.

## Discussion

In this study, we report five main findings. First, we have successfully generated GalNAc-conjugated siRNA duplexes of MASP-1 and MASP-2, and these duplexes exhibit a low IC50 for liver-targeted delivery. Second, these duplexes specifically targeted liver MASP-1 and MASP-2 genes and robustly silence their expression in the liver, leading to systemic depletion of these proteins. Our data confirm that MASP-1 and MASP-2 proteases are generated by the liver and consequently there is little extrahepatic generation of these proteases to effect disease phenotype locally in the joints. Furthermore, targeting of MASP-2 expression inhibited C4b deposition on mannan and therefore functionally inhibited the LP. Third, there was no significant effect of MASP-1 inhibition on CAIA in mice, whereas silencing of MASP-2 via RNAi inhibition partially attenuated arthritis in mice. Four, MASP-1 and MASP-2 may control the AP, possibly by regulating along with MBL the transcription of FD under normal physiological conditions. Finally, our MST data shows that MBL directly interact with TLR4, consistent with the observations of previous laboratories (67, 68).

The role of MASP-1 and MASP-2 proteases in arthritis has been unknown. The most abundant MASP in serum is MASP-1 (69, 70). MASP-1 has been shown to be predominately generated by the liver (36, 38, 71, 72), and it can, therefore, be targeted by RNAi using GalNAc-conjugated siRNAs due to the specific affinity of GalNAc for ASPGR (27, 36, 57). We found that MASP-1 silencing by RNAi had no significant effect on CAIA even with more than 95% silencing at the transcriptional level (in liver) as well as reduction in protein levels (in circulation; Figures 3, 7, 4B). As this gene also produces MASP-3, which is important in the induction of CAIA (40), any effect of this targeting in MASP-3 was not sufficient to affect disease generation. The minor non-significant decrease we have seen might be due to the inhibition of LPS-induced AP by MASP-1 inhibition. If so then it will be consistent with recently published studies showing an unexpected link between the LP and AP (46). We asked the question: why there was no significant decrease in the disease activity even by >95% inhibition of MASP-1 protease systemically? This might be related to difference in the importance of MASP-1 for the AP in *in vitro* and *in vivo* situations. Moreover, disease is dependent on the dose of anti-CII abs but not LPS (13) for LPS injected mice do not develop arthritis, but even if they do it is mild and transient lasting for couple of days (13). CAIA is dependent on the AP (61) and mice lacking C3, FB and FD were somewhat resistance to CAIA (40, 60, 61). Mice lacking MASP-1/3, i.e., lacking both MASP-1 and MASP-3 were also resistance to CAIA (40). The synergistic role of MASP-1 and MASP-2 in RA can't be ruled out, since MASP-1 activates MASP-2 to activate the LP (29). The above mentioned small, non-significant decrease due to MASP-1 silencing in CAIA we have seen might also be due to the delayed activation of the LP MASP-2 by inhibiting MASP-1 for MASP-2 plays an important role to activate the AP via C4 bypass mechanism (62). We have not tested the role of MASP-1 in zymosan-induced arthritis (ZIA) and one can draw inferences that these mice will not be protected based on the above study (46). Furthermore, serum naturally have antibodies to yeast so it can complicate the interpretation by activation antibody-dependent mechanisms such as the CP.

CAIA is dependent not only on the AP but also on generation of C5a. Normally the AP contribute 80–95% of complement activation (73). Mice lacking C5 or treated with GalNAc-C5-siRNA or anti-C5 inhibitory antibody do not develop arthritis (15, 16, 74) despite the presence of MASP-1 and MASP-2. We also found no effect of MASP-1 and MASP-2 silencing on the expression of C5 in the liver (Figure 8H). These data confirm that MASP-1 and MASP-2 inhibition have no effect on the liver expression of downstream complement components, which are essential for the precipitation of disease in the joints. As MASP-1 or MASP-2 is knocked down, the effect of LPS on IL-1β is blunted. As MASP-1 and MASP2 is knocked down, the effect of LPS on IL-1β is completely blocked, indicating that the MASPs are also important for the inflammatory response against LPS. Again, these cytokines data are consistent with CDA in mice treated with MASP-1 or MASP-2 siRNAs (Figure 3A).

Here, we found that nearly complete silencing of MASP-2 expression by RNAi in the liver using GalNAc-MASP-2-siRNA leads to a partial but significant decrease in the CDA (Figure 3A). These results were consistent with our previous study in which mice lacking MASP-2/sMAp were also partially protected from CAIA (62). Interestingly, we have generated the same phenotype in WT mice as seen in *MASP-2/sMAp*^−/−^ mice by injecting GalNAc-MASP-2-siRNA duplex, i.e., nearly complete elimination of MASP-2/sMAp proteins in the circulation (Figure 5B). In this study we could not differentiate between the effects of MASP-2 and sMAp since we have silenced both and the function of sMAp is unknown. The duplex of GalNAc-MASP-2 silenced both MASP-2 and sMAp, therefore, created an identical phenotype to the *MASP-2/sMAp*^−/−^ mice (Figures 5A,B). Thus, liver-targeted inhibition of MASP-2 might be beneficial clinically to treat many ischemia reperfusion injuries (IRI) due to their dependency on MASP-2. In fact, MASP-2 has been shown to play an important role in many IRI mouse models such as myocardial infarction, gastrointestinal IRI, and cerebral IRI because mice lacking MBL or MASP-2 were protected but not mice lacking C4 (75). In these studies it has been shown that MASP-2-dependent C4 bypass pathway activated the AP and played an important role in IRI (75). But none of these studies, have explored the mechanism(s) that leads to MASP-2-dependent C4-bypass activation of the AP and which component of the AP is involved or regulated? One study has shown that MASP-2 can directly cleave C3 without involvement of C4 or C2 (30) but still it is not enough to perpetuate AP activation. Here we report for the first time that MASP-2 might directly regulate the AP by further regulating the transcription of FD in adipose tissue to generate a feedback loop for FD. FD is predominately generated by adipocytes (38, 40), and adipose tissue is present in or around all vital organs of the body including liver and joints (data not shown). Mice lacking fat or FD have defective AP (76) identical to *MASP-1/3*^−/−^ mice or *MASP-3*^−/−^ *mice* (40, 41). Moreover, mice lacking FD or *MBL A/C/FD*^−/−^ mice are totally resistance to arthritis (59).

**Figure 13:**
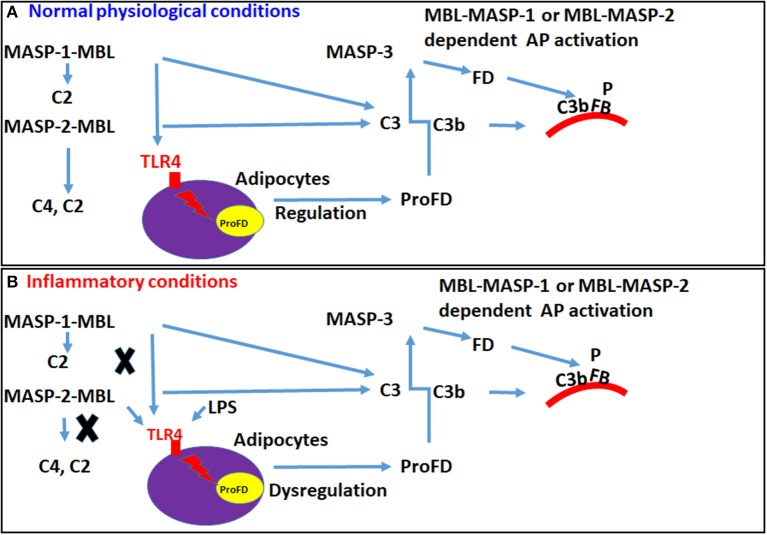
A hypothetical model in mouse showing how MBL or conjugates of MBL-MASP-1 or MBL-MASP-2 can regulate the transcription of FD to regulate the activation of the AP via TLR4 receptors. Circulating MBL alone or MBL-MASP-1 or MBL-MASP-2 complexes can directly interact with disguised TLR4 on adipocytes to activate the complement system via enhancing the expression of FD to regulate the AP pathway. **(A)** MBL or MBL-MASP-1 or MBL-MASP-2 conjugates under normal physiological conditions can bind to the TLR4 and regulate the expression of FD but MASP-2 might be dominant. **(B)** In contrast, under inflammatory conditions, MBL or MBL-MASP-1 or MBL-MASP-2 conjugates might be displaced by the LPS and modulate the transcription of FD through TLR4 receptors.

To further explore mechanisms by which MASP-1 and MASP-2 regulate the transcription of FD, we found that during homeostasis there was a significant decrease of 72% in the expression FD in the liver of mice injected simultaneously with GalNac-MASP-1 plus GalNac-MASP-2 duplexes vs. singly injected mice (Figure 8F). Interestingly, with this combined treatment, there was no effect on expression of C3, FB, and C5 in the liver (Figures 8D,E,H). No decrease in Properdin expression was seen with combined silencing of MASP-1 and MASP-2 (Figure 8G). Properdin is known to stabilize AP-C3 convertase, indicating that MASP-1 and MASP-2 contributes the AP by sustaining the expression of both FD and properdin. Overall these data showed that the conjugates of both MBL-MASP-1 and MBL- MASP-2 might be directly regulating the transcription of FD. Our *in vivo* data show that MASP-1 and 2 regulate FD transcription under normal physiological conditions but not under inflammatory conditions (Figure 8F). An inflammatory conditions in these mice were confirmed by measure of IL-1β and TNF-α in the liver subsequently after silencing of MASP-1 and MASP-2 and LPS injection (Figures 9A,B). We hypothesize that regulation of transcription of FD is switched under inflammatory conditions from MASP-1 and MASP-2 to something else, such as LPS. LPS is a ligand for TLR4 receptors and also competes with MBL for its binding with TLR4 (67, 68). Thus, circulating complexes of MBL-MASP-1 or MBL-MASP-2 or MBL might be replaced with LPS while transitioning from normal to inflammatory conditions. MBL, a prototypical pattern recognition molecule can also modify the inflammatory response during bacterial and viral infections. MBL could suppress LPS-induced TNF-α and IL-12 production in THP-1 cells and monocyte-derived dendritic cells by inhibiting LPS-induced NF-kB DNA binding and translocation (68). We hypothesize that MBL-MASP-1 or MASP-2 conjugates can control the transcription of FD in liver under normal physiological conditions (Figure 13). A limitation of our study is that we have not examined the expression of FD in the liver of *MBL*^−/−^ and *MASP-2*^−/−^ mice and this is due to the non-availability of these mice from any commercial source at the time these data were collected. We have also not used complexes of MBL-MASP-1 or MBL-MASP-2 due to the autocatalytic nature of these enzymes. However, transient knockdown may provide a more representative picture if the importance of MASP-2, as these mice have not had the same opportunity to develop compensatory mechanisms as of *MASP-2*^−/−^ mice.

We confirmed by MST a direct binding between human recombinant MBL and human recombinant TLR4 with an equilibrium dissociation constant (K_d_) of 907.28 nM concentration (Figure 11). To further provide the evidence that interactions between MBL-MASPs and TLR4 can occur, *in vivo*, we found primary adipocytes from mouse adipose tissue injected with LPS have reasonably good expression of TLR4 but a huge expression of FD (data not shown). Conversely, differentiated 3T3L1 cells, which develops adipocyte-like morphology, have high levels of TLR4 but low levels of FD expression (Figure 12). Interestingly, in these differentiated 3T3 cells, with MBL or LPS treatment, TLR4 expression decreases but FD expression increases (Figure 12) again suggesting a link between LPS, TLR4, and FD transcription (Figure 13).

Overall, in this study, we have generated highly potent and specific MASP-1 and MASP-2 siRNA duplexes, especially targeting liver for a longer time with an excellent clinical therapeutic potential. These duplexes can be tested further in various mouse models of diseases involving MASP-1 and MASP-2 such as IgA Nephropathy (77) and experimental pneumococcal meningitis (78) to understand the contribution of liver MASP1 and MASP2 to disease pathology in conditions where LP activations has been implicated. Humanized MASP-2 duplexes can also be developed and tested. We think that our MASP-2 siRNA duplexes present another therapeutic option not only for arthritis but for above mentioned diseases and it will be worth to explore further.

## Data Availability Statement

The datasets generated for this study are available on request to the corresponding author.

## Ethics Statement

The animal study was reviewed and approved by IACUC UC Denver.

## Author Contributions

VH and NB planned the strategy to conjugate GalNAc with MASP-1 and MASP-2 targeting liver, discussed all analyzed data, analyzed all data, and wrote all parts of the manuscript. NB also performed all *in vivo* studies. AB and DD synthesized, conjugated, and tested MASP-1 or MASP-2 duplexes. RS, NH, and JR helped Western blot analysis, *in vitro* and *in vivo* studies. JD and PG helped in designing the experiment and provided valuable reagents and also made valuable suggestions for the discussion. JL performed MBL-TLR-4 binding experiments. AH, RP, and ST performed MASPs related ELISA and Western blots. RP also performed *in vivo* studies along with NB. All authors read this manuscript and made valuable suggestions.

### Conflict of Interest

The authors declare that this study received funding from Alnylam Pharmaceuticals Inc. as a contract to NB. The funder had the following involvement with the study: synthesizing and conjugating duplexes of MASP-1 and MASP-2 with GalNAc. NB is seeking patent protection to use these conjugates for the treatment of rheumatoid arthritis and other complement mediated diseases. The remaining authors declare that the research was conducted in the absence of any commercial or financial relationships that could be construed as a potential conflict of interest.

## Abbreviations

CDA: clinical disease activity
AP: alternative pathway
anti-CCP: anti-cyclic citrullinated peptide
ACPA: anti-citrullinated protein antibodies
CAIA: collagen antibody-induced arthritis
CP: classical pathway
LP: lectin pathway
MBL: Mannan-binding lectin
WT: wild type
GalNAc: N-acetylgalactosamine
ASGPR: Asialoglycoprotein Receptor
rHuC4: recombinant human complement protein 4
proFD: profactor D
FD: factor D
MASP-1: mannan-binding lectin-associated serine protease-1
MASP-2: mannan-binding lectin-associated serine protease-2
MASP-3: mannan-binding lectin-associated serine protease-3
MAp19: Mannan-binding lectin-associated protein of 19 kDa
MAp44: Mannan-binding lectin-associated protein of 44 kDa [a.k.a MBL/ficolin/CL-11–associated protein-1, (MAP-1)]
siRNA: small interfering ribonucleic acid
Luc: Luciferase
mAb: monoclonal antibody
TLR4: Toll-like Receptor 4
LPS: Lipopolysacchride.
